# An intelligent diagnostic method for porcine gastrointestinal infectious diseases based on multimodal AI and large language model

**DOI:** 10.3389/fvets.2025.1660745

**Published:** 2025-09-05

**Authors:** Haiyan Wen, Hongtao Shi, Jiashang Yu, Zhaobin Fan, Haicheng Dai, Lili Jiang, Qinye Song

**Affiliations:** ^1^College of Veterinary Medicine, Hebei Agricultural University, Baoding, China; ^2^College of Pharmacy, Heze University, Heze, China; ^3^School of Science and Information Science, Qingdao Agricultural University, Qingdao, China; ^4^College of Mathematics and Statistics, Heze University, Heze, China; ^5^Rizhao Jiacheng Animal Health Products Co., Ltd, Rizhao, China

**Keywords:** porcine gastrointestinal infectious diseases, multimodal, artificial intelligence, large language model, Mask R-CNN, machine learning

## Abstract

The swine farming industry, a key pillar of Chinese animal husbandry, faces significant challenges due to frequent outbreaks of porcine gastrointestinal infectious diseases (PGID). Traditional diagnostic methods reliant on human expertise suffer from low efficiency, high subjectivity, and poor accuracy. To address these issues, this paper proposes a multimodal diagnostic method based on artificial intelligence (AI) and large language model (LLM) for six common types of PGID. In this method, ChatGPT and image augmentation techniques were first used to expand the dataset. Next, the Multi-scale TextCNN (MS-TextCNN) model was employed to capture multi-granularity semantic features from text. Subsequently, an improved Mask R-CNN model was applied to segment small intestine lesion regions, after which seven convolutional neural network (CNN) models were used to classify the segmented images. Finally, five machine learning models were utilized for multimodal classification diagnosis. Experimental results demonstrate that the multimodal diagnostic model can accurately identify six common types of PGID. This study provides an efficient and accurate intelligent solution for diagnosing PGID and demonstrates superior performance compared with single-modality methods.

## 1 Introduction

The swine farming industry is a crucial component of Chinese animal husbandry. In 2024, China’s pork production reached 57.06 million tons, accounting for 59.05% of the total output of pork, beef, mutton, and poultry (1). The swine farming industry not only plays a vital role in ensuring the safe supply of meat but is also a significant industry related to national economic and social welfare, holding a pivotal position in Chinese agricultural production. During the swine farming process, various diseases frequently occur, with digestive tract infectious diseases being the most common and severe, representing one of the primary causes of piglet mortality (2). Economic losses in swine farms due to digestive tract infectious diseases exceed 10 billion yuan annually, causing substantial financial damage to the swine farming industry (3, 4). Early, rapid, and accurate diagnosis is critical for disease prevention and control in swine.

Traditional clinical diagnostic methods for PGID primarily rely on observing clinical symptoms, pathological changes, and epidemiological data to make preliminary diagnoses, or make confirmed diagnoses on some diseases that present typical and characteristic clinical signs. These methods heavily depend on the expertise and experience of frontline veterinarians, suffering from strong subjectivity, low efficiency, and poor accuracy. Additionally, the specialized skills and experience of veterinary experts are difficult to replicate quickly, leading to a shortage of qualified frontline veterinarians. In contrast, laboratory diagnostic methods utilize advanced detection technologies and sophisticated equipment to enable early diagnosis with high accuracy and robustness, making them one of the most commonly used and effective approaches for diagnosing swine diseases. Among these, multiplex quantitative PCR (qPCR) and enzyme-linked immunosorbent assay (ELISA) are widely applied in diagnosing PGID. Chen et al. developed a triplex qPCR for detecting porcine transmissible gastroenteritis virus, porcine epidemic diarrhea virus, and porcine delta coronavirus, achieving a clinical sample detection concordance rate of approximately 95% (5). Yang et al. established an indirect ELISA using the COE protein of porcine epidemic diarrhea virus expressed in Pichia pastoris as the coating antigen to detect antibodies against porcine epidemic diarrhea in serum, with a detection concordance rate of up to 99.4% (6). Although PCR and ELISA can achieve high diagnostic rates, these methods are complex, time-consuming, costly, and require specialized equipment and trained personnel to perform.

In recent years, with the rapid development of AI and image processing technologies, image recognition techniques based on deep learning have been widely applied in animal disease diagnosis (7–9). Kittichai et al. proposed an automated tool based on deep neural networks and image retrieval procedures for identifying Anaplasmosis, a common livestock disease, in microscopic images (10). This method, utilizing the ResNeXt-50 model combined with the Triplet-Margin loss function, achieved an accuracy of 91.30% and a specificity of 92.83%. Muhammad Saqib et al. introduced a deep learning approach using the MobileNetV2 model and RMSprop optimizer for diagnosing lumpy skin disease in cattle (11). This method demonstrated an accuracy of up to 95%, surpassing existing benchmark methods by 4–10%. Yu et al. developed a deep learning model based on the YOLOv8 detection algorithm, utilizing kidney ultrasound images to classify the International Renal Interest Society (IRIS) stages of chronic kidney disease in dogs (12). This model performed best in distinguishing IRIS stage 3 and above in canine chronic kidney disease, achieving an accuracy of 85%, significantly outperforming the 48–62% accuracy of veterinary imaging experts. Buric et al. employed a U-Net architecture combined with backbone networks such as VGG, ResNet, Inception, and EfficientNet for diagnosing various canine ophthalmic diseases (13). This model exhibited strong reliability, with an Intersection over Union score exceeding 80%, demonstrating high accuracy in the segmentation and diagnosis of canine eye diseases. Although these methods have shown significant success in animal disease diagnosis, their direct application to diagnosing porcine digestive tract remains challenging. The diagnosis of PGID is highly complex, requiring not only the identification of small intestine lesion characteristics from anatomical images but also the integration of textual case information for comprehensive analysis, involving the collaborative processing of multimodal image and text data. Currently, research on multimodal diagnostic techniques is primarily focused on human diseases (14–17), with no related academic reports in the field of PGID diagnosis.

Moreover, due to the sensitive nature of swine disease case information, which involves the interests of farms and the stability of the industry, collecting cases of PGID is challenging, leading to insufficient sample sizes for disease case data. This, in turn, causes issues such as low accuracy and overfitting in diagnostic models. Data augmentation is a key technology for addressing this problem, but traditional text augmentation methods (e.g., synonym replacement, word embeddings) have limited effectiveness. As an LLM, ChatGPT, with its powerful text generation and semantic understanding capabilities, excels in data augmentation, effectively tackling the challenges of insufficient training data and limited diversity. Dai et al. proposed the AugGPT method, which prompts ChatGPT to perform multiple rewrites of sentences, generating semantically similar but diversely expressed samples, significantly improving the accuracy and sample distribution diversity in text classification tasks (18). Fang et al. utilized ChatGPT to generate synthetic text, enhancing the compositional generalization ability of open-intent detection models and improving their capability to handle unseen data (19). Han et al. employed ChatGPT to generate synthetic data to reduce model bias, designing two strategies: targeted prompts and general prompts. The former is more effective but requires predefined bias types, while the latter is more broadly applicable (20). These methods provide viable pathways for text data augmentation in the diagnosis of PGID.

Here, a multimodal AI and LLM-based method was proposed for diagnosing PGID. The method first employs LLMs and image augmentation techniques to enhance case sample data, then uses a MS-TextCNN to extract text features from case reports and adopts an improved Mask R-CNN combined with a CNN classification model to identify small intestine lesion features, and finally utilizes a machine learning model to perform disease classification and diagnosis based on multimodal text and image features, which will effectively address the issue of insufficient text data, and achieve high-precision disease diagnosis.

## 2 Materials and methods

**Figure 1:**
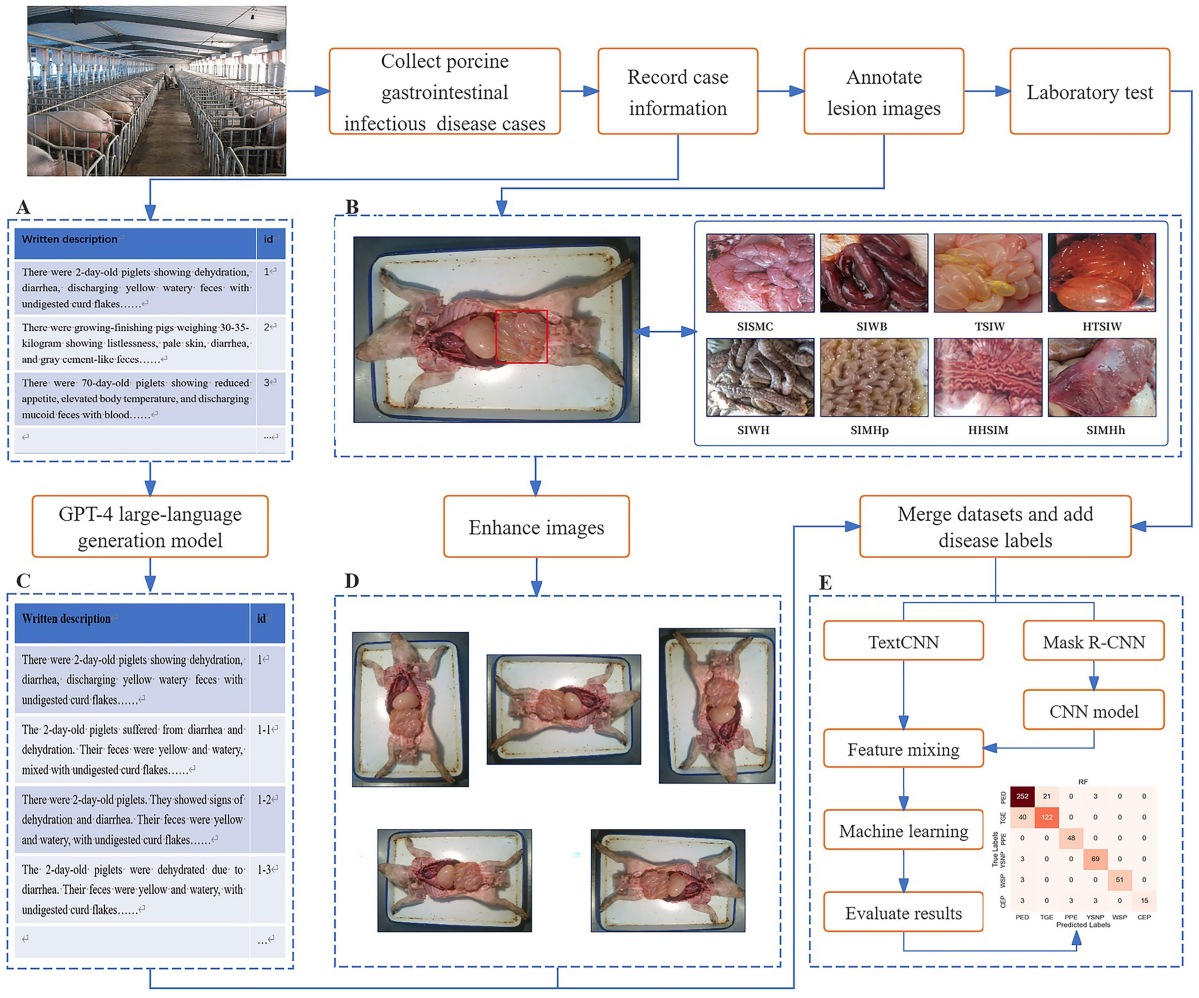
Flow chart of porcine gastrointestinal infectious diseases diagnosis by multimodal AI and LLM.

The experimental workflow for diagnosing PGID using multimodal AI in this study is illustrated in Figure 1. Step A: Constructed a text dataset of PGID case information. This involved using text to describe the onset details of PGID cases, including age at onset, season of onset, disease progression, clinical signs, appetite status, and fecal characteristics. Step B: Constructed and annotated a dataset of swine anatomical images. Domain experts manually annotated the small intestine lesion regions in the anatomical images and assigned classification labels based on eight distinct small intestine lesion characteristics. Step C: Augmented the text dataset of PGID case information. Using ChatGPT-4, each text description was augmented to generate five new text samples that were semantically consistent but vary in expression style. Step D: Augmented the swine anatomical image dataset. New swine anatomical images were generated using rotation (90°, 180°, and 270°) and mirroring (horizontal and vertical), producing five new images per original image. Step E: Implemented disease prediction based on multimodal feature fusion for PGID. The augmented text and image datasets were merged as multi-source data, and each case was labeled with a disease tag based on laboratory disease detection results. MS-TextCNN and Mask R-CNN + CNN branch networks were constructed to extract text and image features, respectively. The extracted features are concatenated, feature-level fusion is performed, machine learning classification is performed, and the classification results are evaluated.

### 2.1 Data collection and preprocessing

#### 2.1.1 Dataset

The swine disease data used in this study were sourced from the Animal Disease Research Institute of Heze University, collected from July to December 2023. The dataset comprised 106 confirmed cases of swine disease, covering 6 common types of PGID: porcine epidemic diarrhea (PED), transmissible gastroenteritis of pigs (TGE), porcine proliferative enteropathy (PPE), yellow scour of newborn piglets (YSNP), white scour of piglets (WSP), and clostridial enteritis of piglets (CEP). Details are provided in Table 1.

**Table 1:** Dataset details of PGID.

Disease type	Disease description	Number
PED	Porcine epidemic diarrhea, caused by *porcine epidemic diarrhea virus*, characterized by watery diarrhea and vomiting (46)	46
TGE	Transmissible gastroenteritis of pigs, caused by *transmissible gastroenteritis virus*, characterized by vomiting, severe diarrhea, and high mortality in 2–3-week-old piglets (47)	27
PPE	Porcine proliferative enteropathy, caused by *Lawsonia intracellularis*, characterized by proliferation of crypt epithelial cells in the ileum and colon, leading to thickened intestinal mucosa (48)	8
YSNP	Yellow scour of newborn piglets, caused by pathogenic *Escherichia coli*, characterized by severe diarrhea, yellow watery feces, and rapid death (49)	12
WSP	White scour of piglets, also caused by pathogenic *Escherichia coli*, characterized by milky-white or grayish pasty feces (49)	9
CEP	Clostridial enteritis of piglets, caused by *Clostridium perfringens* type C, characterized by red feces, small intestine mucosal hemorrhage and necrosis, with rapid onset, short disease course, and high mortality (4)	4

**Figure 2:**
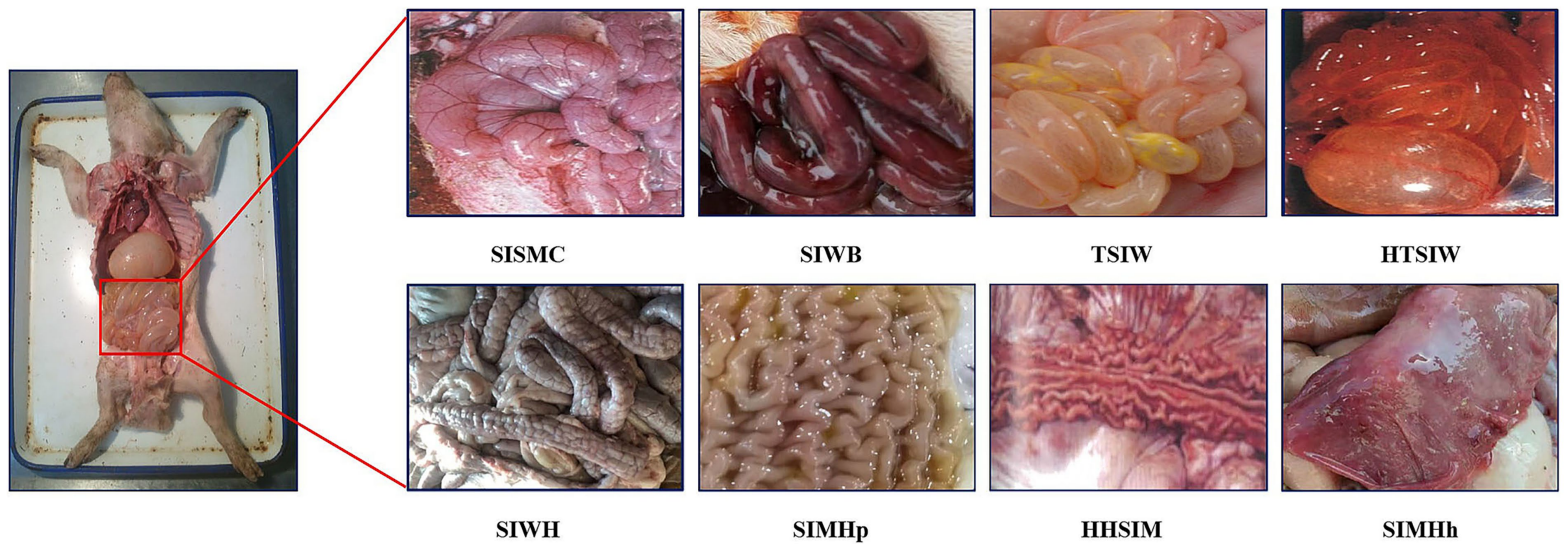
Lesion features in porcine small intestine. SISMC, the serosal layer shows dilated and engorged capillaries, appearing bright red with prominent texture; SIWB, Damaged mucosal capillaries cause blood to enter the intestinal lumen, with affected segments appearing dark red; TSIW, the intestinal wall becomes thin, even transparent, losing its original toughness and elasticity; HTSIW, the intestinal wall appears red, becomes thin and transparent, and loses its toughness; SIWH, the intestinal wall thickens, resembling a soft hose, with a rough or granular surface; SIMHp, the mucosal surface thickens, exhibiting longitudinal and transverse wrinkles with an uneven surface; HHSIM, the mucosa appears red or dark red, with thickened layers, a rough surface, and accompanying wrinkles; SIMHh, the mucosal surface shows bright red or dark red dotted, patchy, or diffuse hemorrhages.

Each case included data in two different formats: a textual description of the swine’s disease onset information and anatomical images of the swine containing small intestine lesion regions. The textual description of the onset information covered details such as age at onset, season of onset, disease progression, clinical signs, appetite status, and fecal characteristics. The swine anatomical images included the lesion regions of the small intestine, with lesion characteristics primarily classified into 8 categories: small intestinal serous membrane congestion (SISMC), small intestinal wall bleeding (SIWB), hemorrhage and thinning of small intestinal wall (HTSIW), thinning of small intestinal wall (TSIW), small intestinal wall hyperplasia (SIWH), small intestinal mucosal hyperplasia (SIMHp), hemorrhage and hyperplasia of small intestinal mucosa (HHSIM), and small intestinal mucosal hemorrhage (SIMHh) (see Figure 2 for details).

#### 2.1.2 Data preprocessing

To mitigate the issues of classification instability and overfitting caused by insufficient data samples, this section applied data augmentation to both the textual descriptions of swine disease case information and the anatomical images of swine. For text augmentation, ChatGPT was utilized for natural language generation. ChatGPT, built on the GPT-4 architecture, is an autoregressive language model with a core Transformer decoder structure. Trained on large-scale corpora through unsupervised pre-training, it possesses robust semantic modeling and language generation capabilities. Without requiring fine-tuning, ChatGPT can generate high-quality text samples that maintain semantic consistency but vary in expression style through input prompts. The conditional probability formula of ChatGPT’s language model is presented in Equation 1:


(1) $$P(x)=\prod_{i=1}^{m}P(s_{i}|s_{1},s_{2},...s_{i-1})$$

Where $x=(s_{1},s_{2},\ldots ,s_{m})$ represents the generated text sequence, and $s_{i}$ is the i-th word or token. By maximizing this conditional probability distribution, ChatGPT generates complete sentences, achieving both semantic preservation and diversity in expression. For each original clinical case description, a prompt (“Based on the text above, generate 5 sentences that have the same meaning but different expressions.”) was constructed and inputted into ChatGPT to generate five semantically consistent but stylistically diverse text samples, thereby expanding the dataset. Through this method, the original text samples were expanded from 106 to 636, significantly enhancing corpus richness and linguistic variability, thus improving the model’s ability to recognize and understand different expression styles.

Image augmentation employs various geometric transformation operations, including rotation by 90°, 180°, and 270°, as well as horizontal and vertical mirroring. These methods significantly increased the diversity of image samples while ensuring that the semantic characteristics of small intestine lesions remained unchanged. The augmented image dataset was also expanded to 6 times the original size, improving the model’ generalization ability across different image angles, orientations, and visual perturbations. Image augmentation not only increased the scale of training data but also provided a more comprehensive feature representation space during training, enhancing the robust identification of lesion regions.

Sequencely, the Labelme software was used to manually annotate the augmented images, accurately delineating the boundaries of small intestinal lesion areas and explicitly labeling their lesion types. During the model testing phase, 5-fold cross-validation was employed for objective evaluation to comprehensively assess the model’s generalization ability and stability.

### 2.2 Multi-scale TextCNN

TextCNN is a CNN-based text classification model widely used in natural language processing tasks (21). Its core principle involves representing text as a word embedding matrix, capturing local semantic features (e.g., n-gram patterns) through convolutional filters, retaining prominent features via max-pooling, and outputting classification results through fully connected layers and a softmax layer (22). TextCNN is renowned for its efficient feature extraction and robustness in handling short texts, making it suitable for classifying case information descriptions in porcine digestive tract infectious disease diagnosis.

**Figure 3:**
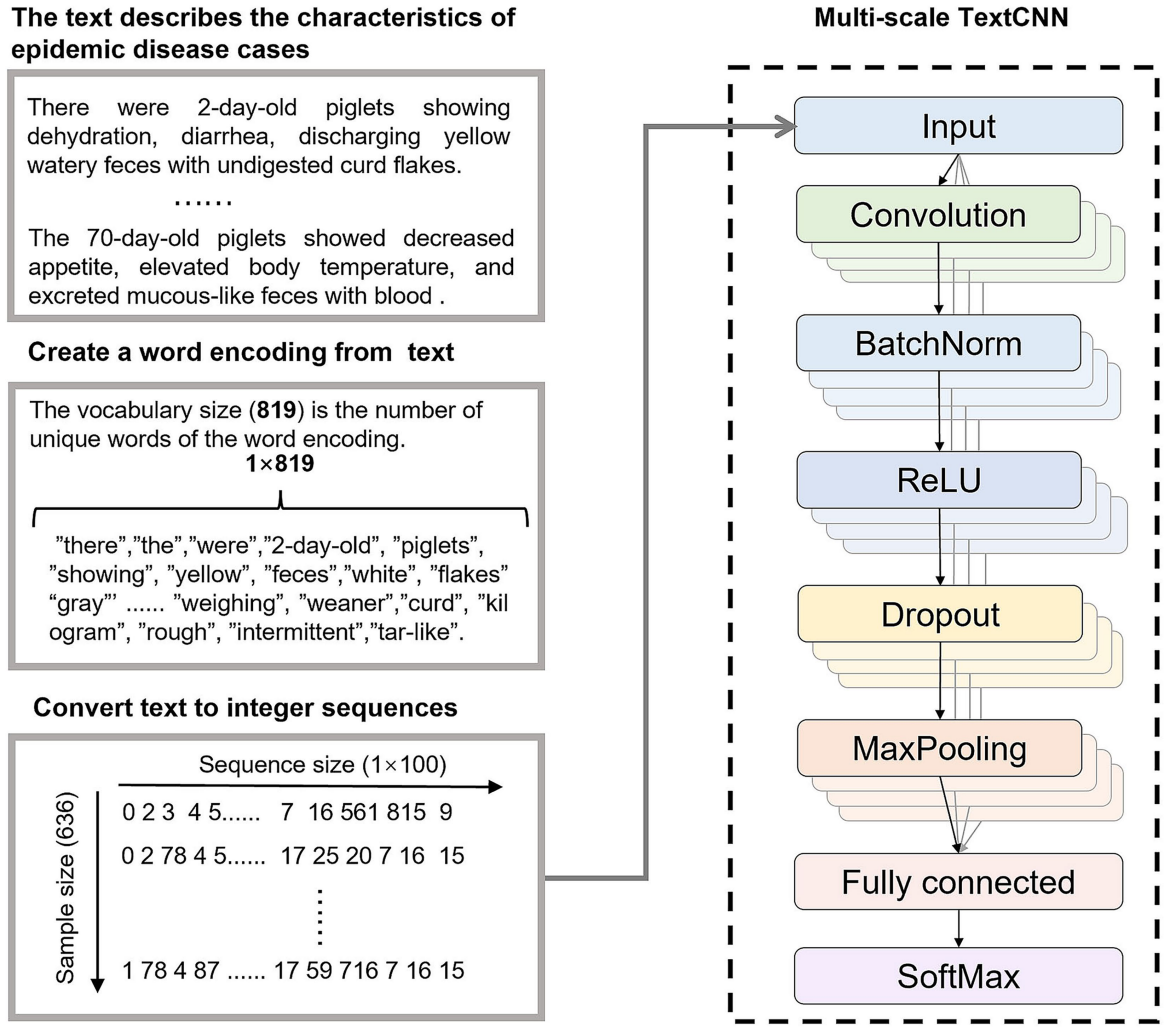
Disease diagnosis based on MS-TextCNN.

The MS-TextCNN proposed in this paper (shown in Figure 3) enhanced the modeling capability for complex texts by introducing convolutional kernels of multiple sizes to extract semantic features of varying lengths in parallel. In the context of porcine gastrointestinal infectious disease diagnosis, the model took 100-dimensional word embeddings as input, employed 128 multi-scale filters, and integrated batch normalization, Rectified Linear Unit (ReLU) activation, Dropout, and global max-pooling to output a 6-class classification.

### 2.3 Improved Mask R-CNN

In practical diagnosis, small intestine lesion regions typically occupy only a small portion of anatomical images (as shown in Figure 2). Directly using a CNN model for whole-image classification is susceptible to interference from irrelevant background, which affects recognition accuracy. Therefore, it is necessary to first use an image detection model (e.g., Mask R-CNN) to locate and extract lesion regions before performing image classification to enhance the model’s recognition performance. Compared to single-stage detection algorithms like YOLO, Mask R-CNN employs a two-stage detection mechanism, making it superior in small target detection and high-precision tasks.

To improve the recognition and segmentation accuracy of small intestine lesion regions, this study optimized the original Mask R-CNN model by incorporating the High-Resolution Network (HRNet) as the backbone network and embedding the Convolutional Block Attention Module (CBAM) attention mechanism during the feature extraction stage. HRNet can extract rich semantic features while maintaining spatial resolution, effectively preserving detailed information of lesion regions. CBAM, through its channel and spatial attention mechanisms, guides the model to focus on more discriminative feature regions. This improved structure enhanced the model’s perception and segmentation accuracy for small target lesion regions while retaining Mask R-CNN’s multi-task detection and segmentation capabilities.

**Figure 4:**
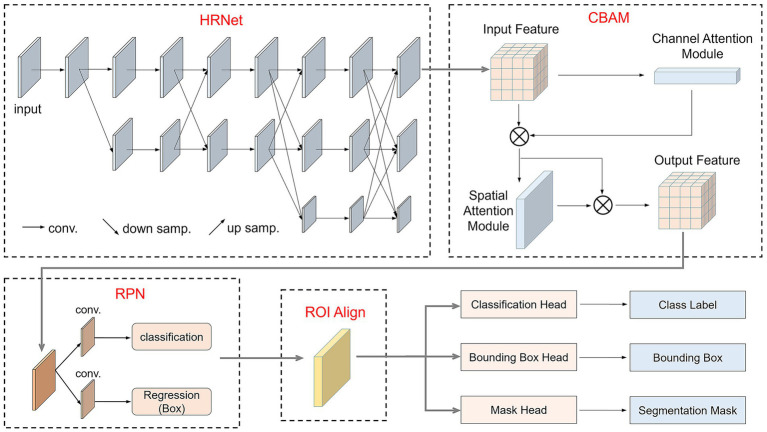
Structure of the improved Mask R-CNN.

The improved model, as shown in Figure 4, replaces the original ResNet-FPN backbone with HRNet and incorporates CBAM modules in key output layers to enhance feature representation. After image input, HRNet first extracts multi-scale high-resolution features, which are then enhanced by the CBAM attention module in both channel and spatial dimensions. Subsequently, the Region Proposal Network (RPN) generates candidate bounding boxes on the feature map for preliminary target localization. The candidate regions are aligned using ROIAlign and fed into three branches: a fully connected layer for classification, a regression layer for bounding box regression, and a fully convolutional network (FCN) for mask prediction. The final output includes the category, bounding box, and corresponding pixel-level segmentation mask for each candidate region.

### 2.4 CNN classification models

This study selected 7 classic CNN classification models for classifying segmented images. Alex Krizhevsky’s Convolutional Neural Network (AlexNet) employs large convolutional kernels and overlapping pooling layers, combined with ReLU activation and Dropout techniques, to effectively enhance image feature extraction capabilities, achieving groundbreaking results in the 2012 ImageNet challenge (23). Visual Geometry Group Network (VGGNet) progressively deepens the network using multiple 3 × 3 convolutional layers and pooling layers, capturing rich features from low to high levels, and demonstrates outstanding performance in various image recognition tasks (24). GoogleNet utilizes Inception modules to apply convolutional kernels of different sizes in parallel, capturing multi-scale features, while replacing fully connected layers with global average pooling to reduce computational complexity and improve efficiency (25). Residual Network (ResNet) introduces residual learning and skip connections, enabling effective training of deep networks, addressing the vanishing gradient problem, and excelling in image recognition tasks (26). Dense Convolutional Network (DenseNet) employs dense connections, allowing each layer to receive feature maps from all preceding layers, significantly improving information flow, reducing the vanishing gradient issue, and enhancing model generalization and efficiency (27). EfficientNet is a convolutional neural network architecture that simultaneously balances the depth, width, and resolution of the network through composite coefficients, significantly reducing model parameters and computational complexity while achieving higher accuracy (28). Vision Transformer divides images into patches and models them using a pure Transformer encoder, excelling at capturing global features and demonstrating image recognition performance comparable to or even superior to CNNs when trained on a large scale (29).

### 2.5 Machine learning models

This study selected 5 classic machine learning classification algorithms for the final disease classification. Naive Bayes (NB), based on Bayes’ theorem, calculates class probabilities through feature independence assumptions, making it suitable for high-dimensional sparse data with advantages of efficient computation and ease of implementation (30). K-Nearest Neighbors (KNN) performs classification through majority voting based on sample distances, offering an intuitive and interpretable decision process, particularly suitable for small-scale datasets (31). Support Vector Machine (SVM) constructs a hyperplane by maximizing the classification margin, effectively handling high-dimensional and nonlinear problems, and performs exceptionally well with small sample datasets (32). Random Forest (RF) integrates multiple decision trees, enabling automatic feature selection and reducing overfitting, with strong robustness suitable for complex datasets (33). eXtreme Gradient Boosting (XGBoost) employs a gradient boosting framework with regularization and optimized feature splitting, significantly improving accuracy and efficiency in modeling large-scale datasets and nonlinear relationships (34).

### 2.6 Evaluation metrics

This study adopted accuracy, precision, recall, and F1 score as evaluation metrics for model performance. Accuracy measures the overall classification prediction performance of the model, calculated as shown in Equation 2:


(2) $$\text{Accuracy}=\frac{\mathrm{TP}+\mathrm{TN}}{\mathrm{TP}+\mathrm{TN}+\mathrm{FP}+\mathrm{FN}}$$

Where TP represents true positives (correctly predicted positive samples), TN represents true negatives (correctly predicted negative samples), FP represents false positives (negative samples incorrectly predicted as positive), and FN represents false negatives (positive samples incorrectly predicted as negative). Accuracy reflects the overall correctness of predictions across all sample categories.

Precision focuses on the proportion of true positives among samples predicted as positive, calculated as shown in Equation 3:


(3) $$\text{Precision}=\frac{\mathrm{TP}}{\mathrm{TP}+\mathrm{FP}}$$

A high precision indicates a low false positive rate.

Recall measures the proportion of actual positive samples correctly identified, calculated as shown in Equation 4:


(4) $$\text{Recall}=\frac{\mathrm{TP}}{\mathrm{TP}+\mathrm{FN}}$$

A high recall indicates a low false negative rate.

The F1 score is the weighted harmonic mean of precision and recall, calculated as shown in Equation 5:


(5) $$F1=2\times \frac{\text{Precision}\times \text{Recall}}{\text{Precision}+\text{Recall}}$$

Its value ranges from 0 to 1, with a higher value indicating a better balance between the two types of errors.

### 2.7 Experimental environment setup

The computer used in this study is equipped with an Intel Core i5-12400F CPU, 32GB RAM, and an Nvidia RTX 4060 GPU. During training, the Adam optimizer was used with an initial learning rate of 0.0001, a cross-entropy loss function, a batch size of 32, 200 training epochs, and early stopping set to 30. Experiments confirmed that this was sufficient for effective training.

## 3 Results and analysis

### 3.1 Mask R-CNN detection results

**Figure 5:**
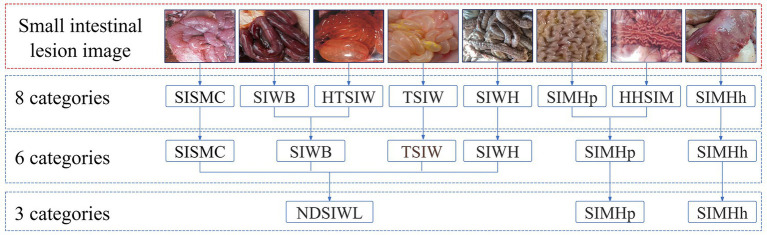
Classification relationship of porcine small intestine lesion image.

This study employed an improved Mask R-CNN network to detect and segment lesion regions in small intestine anatomical images. To optimize the detection performance of Mask R-CNN, three classification methods were used to annotate lesion regions, as shown in Figure 5. The first method was based on eight true lesion characteristics provided by domain experts. The second method merged these into six categories. The third method further consolidated the six categories into three. The choice of classification labels directly impacts Mask R-CNN’s detection performance, and merging similar categories can reduce inter-class uncertainty, thereby improving the model’s recognition performance and generalization ability.

Table 2 presents the detection performance of Mask R-CNN under different classification methods. In the 8-class scenario, except for “TSIW” and “SIWH,” the Precision, Recall, and F1 scores for all other categories were 0, with an overall accuracy (OA) of only 0.0845. This suggests that overly fine-grained category divisions may hinder the model’s ability to effectively identify lesion types. In the 6-class scenario, detection performance improved for some categories, with “SIWB” achieving a Recall of 0.8125 and an F1 score of 0.5226, though the OA remained low at 0.287. In the 3-class scenario, detection performance significantly improved across all categories, particularly for “Non-dissected small intestinal wall lesions (NDSIWL),” where Precision, Recall, and F1 scores reached 0.9277, and OA increased to 0.8202. This indicates that merging similar categories enhances Mask R-CNN’s detection performance and improves the model’s generalization ability.

**Table 2:** Detection results of the improved Mask R-CNN under different classification methods.

Category number	Metric	SISMC /NDSIWL	SIMHh	SIMHp	TSIW	SIWH	SIWB	HTSIW	HHSIM	OA
8	Precision	0	0	0	0.2632	0.2727	0	0	0	0.0845
	Recall	0	0	0	0.3191	0.3000	0	0	0	
	F1	0	0	0	0.2885	0.2857	0	0	0	
6	Precision	1	0	0.75	1	0	0.3852	\	\	0.2870
	Recall	0.1282	0	0.075	0.0426	0	0.8125	\	\	
	F1	0.2273	0	0.1364	0.0816	0	0.5226	\	\	
3	Precision	0.9277	0.5238	0.7333	\	\	\	\	\	0.8202
	Recall	0.9277	0.5500	0.4681	\	\	\	\	\	
	F1	0.9277	0.5366	0.5714	\	\	\	\	\	

To further analyze the recognition performance of Mask R-CNN, Table 3 presents the detection rates under different classification methods to evaluate whether Mask R-CNN successfully detects lesion regions even when it fails to classify them correctly. The results show that in the 8-class scenario, the detection rates for all categories were low, with an overall detection rate of only 0.3521. In the 6-class scenario, the overall detection rate improved to 0.6620, with “SIWB” reaching a detection rate of 0.8281, and other categories also showing improved detection rates, indicating that merging similar categories enhances the ability to detect target regions. In the 3-class scenario, the detection rates for all categories significantly improved, particularly for “NDSIWL” and “SIMHh,” which achieved detection rates of 0.9814 and 0.9000, respectively. The overall detection rate increased to 0.9430, demonstrating that broader category merging significantly enhances the detection capability for lesion regions.

**Table 3:** Detection rate of the improved Mask R-CNN under different classification methods.

Category Number	SISMC /NDSIWL	SIMHh	SIMHp	TSIW	SIWH	SIWB	HTSIW	HHSIM	OA
8	0.2778	0.1875	0.2273	0.4894	0.3000	0.4000	0.5714	0.1667	0.3521
6	0.7436	0.1875	0.4250	0.7234	0.7000	0.8281	\	\	0.6620
3	0.9814	0.9000	0.8298	\	\	\	\	\	0.9430

Combining the results from Tables 2, 3, it is evident that in the 3-class scenario, Mask R-CNN performs best in terms of both recognition accuracy and detection rate. However, the 3-class approach cannot fully describe the true lesion characteristics of the affected regions. Therefore, it is necessary to introduce a CNN network for secondary classification of the segmented images.

### 3.2 CNN classification results

To investigate the impact of different classification granularities, the experiment was conducted with two approaches: 8-class and 6-class classifications, while the 3-classification method was solely used for image segmentation of lesion regions. Classic CNN models described in section 2.4 were trained and tested on the segmented images. The experimental results, as shown in Table 4, indicate that all CNN models achieved higher classification accuracy on the pixel-level segmented images, with the OA of the 6-class approach generally outperforming the 8-class approach. Although the 8-class approach can more finely characterize lesion features, it also increases classification difficulty, resulting in slightly lower accuracy. For example, GoogleNet achieved an accuracy of 96.74% in the 8-class scenario, which further improved to 97.67% in the 6-class scenario. Similarly, DenseNet’s accuracy increased from 95.81 to 96.74%, Vision Transformer’s accuracy increased from 94.88 to 96.74%. This suggests that for fine-grained lesion classification tasks, moderately merging similar categories can reduce the model’s learning difficulty, thereby achieving better classification performance.

**Table 4:** Classification results of improved Mask R-CNN-segmented images by different CNN models.

Models	8 Categories				6 Categories			
	Acc	P	R	F1	Acc	P	R	F1
AlexNet	0.8326	0.8000	0.8189	0.8038	0.9023	0.8867	0.8983	0.8883
DenseNet	0.9581	0.9438	0.9575	0.9500	0.9674	0.9683	0.9617	0.9683
GoogleNet	0.9674	0.9613	0.9675	0.9613	0.9767	0.9717	0.9750	0.9733
ResNet	0.8047	0.7950	0.7750	0.7700	0.8791	0.8441	0.8800	0.8617
VGGNet	0.8279	0.8463	0.7600	0.7500	0.8465	0.8400	0.8250	0.8217
EfficientNet	0.9209	0.9239	0.8937	0.9052	0.9256	0.9170	0.8966	0.9053
Vision Transformer	0.9488	0.9342	0.9571	0.9423	0.9674	0.9659	0.9419	0.9524

### 3.3 Multimodal disease recognition

This study employed five representative machine learning models described in Section 2.5 to evaluate the classification performance of different feature modalities for PGID diagnosis. The classifiers were first applied to the text features (10 dimensions) extracted by MS-TextCNN, with results summarized in Table 5. Among all models, RF achieved the best overall performance, reaching the highest accuracy 0.8479, precision 0.9198, recall 0.8564, and F1 score 0.8870. In contrast, NB performed the worst, with an accuracy of only 0.6261, despite showing relatively high precision 0.7821. The remaining models demonstrated generally good performance but were slightly less accurate and stable compared with RF. These results indicate that text-based clinical features are highly discriminative.

**Table 5:** Classification results of text features by different machine learning models.

Models	Acc	*P*	*R*	F1
NB	0.6261	0.7821	0.7369	0.7588
KNN	0.8132	0.8668	0.8213	0.8434
SVM	0.8399	0.9073	0.8481	0.8767
RF	0.8479	0.9198	0.8564	0.8870
XGBoost	0.8399	0.8798	0.8279	0.8531

Then, the classifiers were applied to the image features (4 dimensions) extracted by Mask R-CNN + CNN under both 8-class and 6-class encodings, and the performance of all classifiers declined considerably, as shown in Table 6. The best results again came from RF, which achieved the highest accuracy 0.5526 for 8-class and 0.5476 for 6-class, with F1 scores around 0.58. At the other extreme, NB remained the weakest, with accuracies of 0.3987 for 8-class and 0.3949 for 6-class, and F1 scores near 0.41. The other models exhibited intermediate performance between the best and worst results, revealing certain limitations relative to the RF model. Overall, the results confirm that compared with text features, image features alone are less discriminative and less reliable for PGID classification.

**Table 6:** Classification results of image features by different machine learning models.

Models	8-Class image features				6-Class image features			
	Acc	*P*	*R*	F1	Acc	*P*	*R*	F1
NB	0.3987	0.4165	0.4052	0.4108	0.3949	0.4168	0.3977	0.4080
KNN	0.4543	0.9126	0.8730	0.8924	0.4568	0.9168	0.8720	0.8826
SVM	0.4697	0.5176	0.4647	0.4897	0.4476	05008	0.4570	0.4769
RF	0.5526	0.5871	0.5731	0.5800	0.5476	05866	0.5687	0.5765
XGBoost	0.5149	0.5357	0.5047	0.5197	0.5079	0.5122	0.4976	0.5121

**Figure 6:**
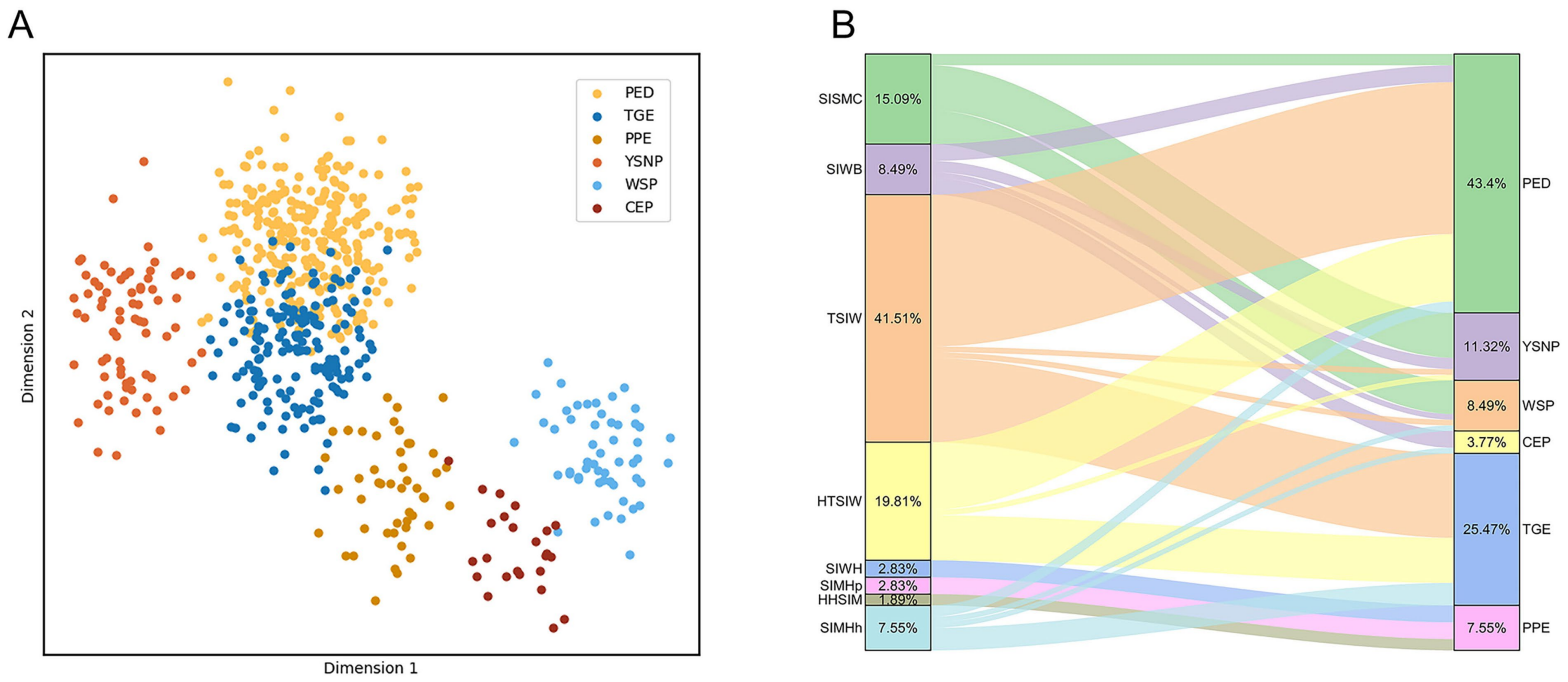
Visualization of single-modal classification effects. **(A)** Visualization of text classification effects. **(B)** Visualization of the mapping between small intestine lesion characteristics and porcine gastrointestinal infectious diseases.

Figure 6 presents the visual results of text and image features. The LDA projection of text features (Figure 6A) reveals substantial overlap between PED and TGE, with blurred boundaries among other categories, which explains the residual misclassifications observed in Table 5 despite the strong performance of text-only models. In contrast, the Sankey diagram of small-intestine lesion features (Figure 6B) illustrates a many-to-many correspondence between lesion traits and PGID classes, thereby clarifying why the image-only models reported in Table 6 performed poorly.

To further improve the recognition performance, this study performed feature-level fusion, combining the text features from MS-TextCNN with the image features from Mask R-CNN + CNN under both 8-class and 6-class encodings. The results are shown in Table 7. Among them, RF achieved the best performance, with accuracies of 87.58% (text + 8-class image features) and 86.47% (text + 6-class image features), outperforming all single-modality baselines. KNN, SVM, and XGBoost also demonstrated high overall accuracies (all >83%), validating the robustness of multimodal fusion, while NB remained the weakest (66.03 and 62.78%). Additionally, most models performed slightly better with the text + 8-class scheme, suggesting that finer-grained image encoding provides richer complementary cues to bagging classification.

**Table 7:** Classification results of combined features by different machine learning models.

Models	Text features + 8-class image features				Text features + 6-class image features			
	Acc	*P*	*R*	F1	Acc	*P*	*R*	F1
NB	0.6603	0.8305	0.8152	0.8228	0.6278	0.7623	0.7749	0.7685
KNN	0.8569	0.9126	0.8730	0.8924	0.8569	0.9168	0.8720	0.8938
SVM	0.8443	0.9158	0.8564	0.8851	0.8455	0.9198	0.8564	0.8870
RF	0.8758	0.9301	0.8754	0.9019	0.8647	0.9249	0.8677	0.8954
XGBoost	0.8381	0.9093	0.8474	0.8773	0.8496	0.8898	0.8376	0.8629

**Figure 7:**
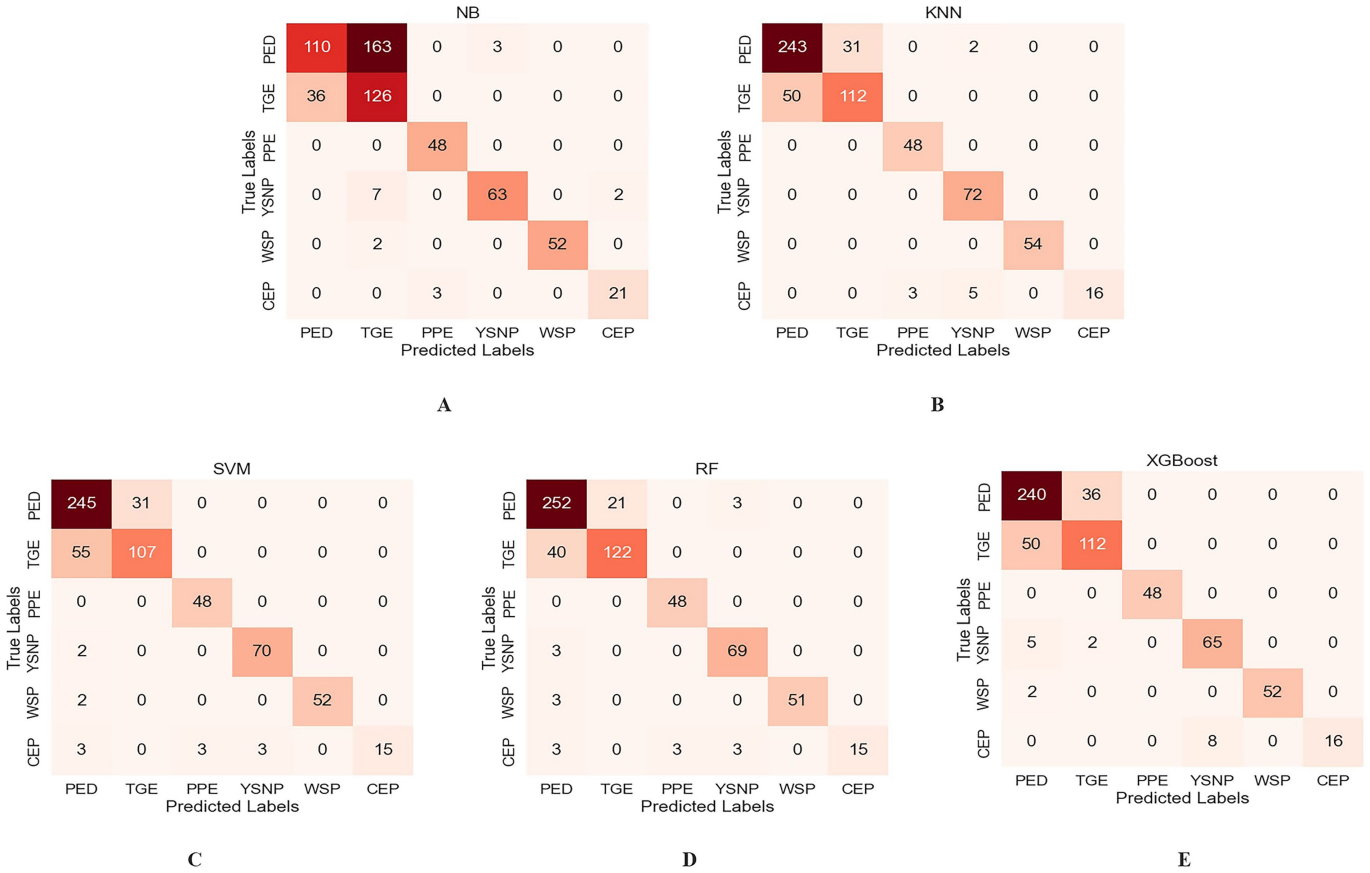
Confusion matrices of different machine learning models. **(A)** NB. **(B)** KNN. **(C)** SVM. **(D)** RF. **(E)** XGBoost.

To more intuitively analyze and compare the recognition performance for each disease, confusion matrices for the classification results of each model under the 8-class image classification scenario were plotted, as shown in Figure 7. The confusion matrices indicate that RF model performed best, with only 21 PED cases misclassified as TGE, achieving a recognition accuracy of 87.58%. In contrast, NB model exhibited the poorest performance, with significant errors in distinguishing PED and TGE, resulting in lower model accuracy. KNN, SVM, and XGBoost models showed improved performance over NB model but did not match the superior accuracy of the RF model. Furthermore, the confusion matrix revealed a severe class imbalance issue, where the rare class CEP showed the lowest recognition performance across most models. As shown in Figure 7, the RF model achieved a recall rate of only 62.5% for CEP. In contrast, the NB model yielded the highest recall for CEP at 87.5%. We further explored common imbalance-handling strategies such as SMOTE (35), ADASYN (36), and AdaBoost (37), but none of them yielded notable improvements for the minority class CEP.

**Figure 8:**
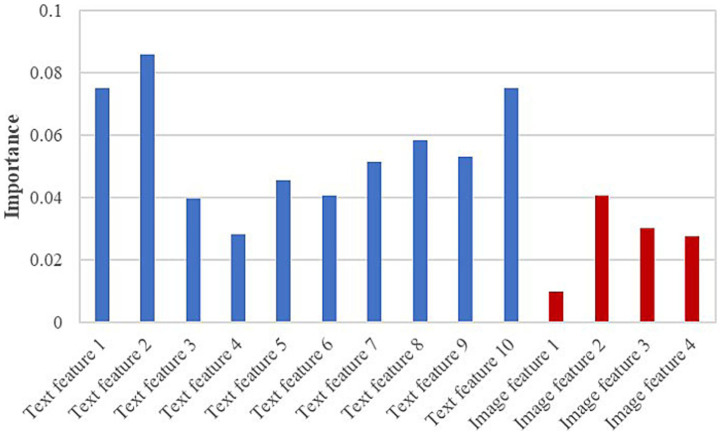
Importance scores for all features used in the RF model.

To further assess the contribution of each feature to the RF model, Figure 8 presents the importance scores of the text features and 8-class image features used in model construction. In the figure, blue bars denote text features and red bars denote image features. The results indicate that text features contribute more substantially to the model than image features. Further examination of the textual descriptions of PED and TGE revealed that some samples share highly similar keywords, which aligns with the partial overlap observed in Figure 6A. Moreover, as shown in Figure 6B, both diseases exhibit TSIW-type and HTSIW-type lesions in the anatomical images of the small intestine. These similarities in textual descriptions and lesion types collectively contribute to the difficulty in distinguishing certain PED and TGE cases.

### 3.4 Comparison with YOLO

YOLO uses attention mechanisms and dynamic convolution to especially improve small object detection, achieving better detection accuracy and computational efficiency (38). To further validate the advantage of the proposed Mask R-CNN in improving the recognition accuracy of CNN networks, YOLO was used to detect swine anatomical images, with results shown in Table 8. It can be observed that under three different classification scenarios, the recognition performance of YOLOv8 is similar to that of the proposed Mask R-CNN model but slightly inferior. YOLOv8 (39) demonstrates slightly better recognition performance than YOLOv12 (40) and YOLOv13 (41), making it the most effective YOLO model under the data conditions of this study.

**Table 8:** Detection results of the YOLO under different classification methods.

Models	Category number	SISMC /NDSIWL	SIMHh	SIMHp	TSIW	SIWH	SIWB	HTSIW	HHSIM	OA
YOLOv8	8	0.2728	0.1851	0.2213	0.4872	0.2965	0.3932	0.5651	0.1619	0.3478
	6	0.7384	0.1794	0.4205	0.7151	0.6944	0.8207	\	\	0.6542
	3	0.9777	0.894	0.8213	\	\	\	\	\	0.9354
YOLOv12	8	0.2688	0.1853	0.2253	0.4700	0.2896	0.3795	0.5538	0.1587	0.3341
	6	0.7136	0.1853	0.4110	0.7133	0.6355	0.8113	\	\	0.6340
	3	0.9654	0.8600	0.7986	\	\	\	\	\	0.9140
YOLOv13	8	0.2758	0.1875	0.2253	0.4864	0.2997	0.3988	0.5567	0.1651	0.3431
	6	0.7349	0.1875	0.4110	0.7213	0.6300	0.8170	\	\	0.6480
	3	0.9724	0.8876	0.8156	\	\	\	\	\	0.9314

The segmented images from YOLO v8 were input into the CNN networks for classification, with experimental results shown in Table 9. Compared to the results in Table 4, the classification accuracy of all CNN models in Table 9 is significantly lower. Among them, DenseNet achieved the highest classification performance, with accuracies of 94.34 and 94.81% for the 8-class and 6-class scenarios, respectively. These results indicate that the classification performance of YOLOv8-segmented images is generally lower than that of Mask R-CNN, demonstrating the superiority of Mask R-CNN’s pixel-level image segmentation technology in classification and recognition tasks.

**Table 9:** Classification results of YoloV8-segmented images by different CNN models.

Models	8 Categories				6 Categories			
	Acc	*P*	*R*	F1	Acc	*P*	*R*	F1
AlexNet	0.7453	0.7475	0.7362	0.7325	0.8443	0.8627	0.7783	0.8050
DenseNet	0.9434	0.9350	0.9625	0.9438	0.9481	0.9517	0.9450	0.9450
GoogleNet	0.9151	0.9225	0.9150	0.9113	0.9434	0.9533	0.9333	0.9417
ResNet	0.7358	0.7100	0.7175	0.7075	0.8302	0.8550	0.8033	0.8217
VGGNet	0.7972	0.8113	0.7475	0.7688	0.8066	0.7783	0.7733	0.7717
EfficientNet	0.8661	0.8355	0.8461	0.8408	0.9023	0.8967	0.8764	0.8864
Vision transformer	0.9017	0.8981	0.8871	0.8926	0.9324	0.9311	0.9246	0.9292

## 4 Discussion

### 4.1 Advantages of multimodal information fusion

In the diagnosis of PGID, traditional single-modal diagnostic methods, whether relying on empirical observation of clinical symptoms or single laboratory testing techniques, have significant limitations (Figure 6). In contrast, this study integrates anatomical images of swine small intestines with disease case information to construct a multimodal diagnostic model, effectively addressing these shortcomings.

Multimodal information fusion integrates information from multiple data sources to achieve complementarity and enhancement, thereby obtaining richer, more comprehensive, and more accurate information, which in turn improves detection performance (42, 43). In this study, image data intuitively present visual features such as the morphology and location of small intestine lesions, while case information includes contextual and symptomatic details such as age at onset, season, and disease progression. These two modalities complement each other, providing a more comprehensive and multidimensional basis for disease diagnosis. At the model level, multimodal feature fusion enables the model to learn associations and complementary information between different modalities, enhancing its ability to represent complex disease characteristics. Experimental results demonstrate that the multimodal recognition model significantly outperforms single-modal models in disease classification accuracy. The RF algorithm, after fusing multimodal features, achieved a diagnostic accuracy of 87.58%, fully highlighting the substantial potential of multimodal information fusion in improving diagnostic accuracy and reliability.

### 4.2 High-quality text generation by LLM

The collection of porcine digestive tract infectious disease cases is challenging, and the limited sample size results in insufficient training data, which is a key factor constraining the performance of diagnostic models. ChatGPT, as an advanced LLM, leverages its powerful semantic understanding and text generation capabilities to produce a large number of semantically consistent but diversely expressed text samples through simple prompt inputs (18). This approach expanded the original text dataset from 106 to 636 samples, significantly enriching the training data.

These high-quality generated texts not only increase data diversity but also enable the model to learn a broader range of linguistic expressions, improving its ability to recognize and understand case information described in different styles. During actual training, models augmented with LLM-generated data exhibited better generalization when processing new, unseen text descriptions, effectively mitigating overfitting issues caused by data scarcity. Additionally, the process of text generation using LLMs requires no complex model fine-tuning, offering an efficient and convenient solution for addressing the issue of insufficient text data in swine disease diagnosis.

### 4.3 Advantages of combining Mask R-CNN with CNN models

In swine anatomical images, small intestine lesion regions often occupy a small portion and are surrounded by complex backgrounds. Directly applying CNN for whole-image classification is prone to interference from irrelevant information, leading to reduced recognition accuracy. Mask R-CNN is a two-stage framework-based model capable of simultaneously performing object detection and instance segmentation (44). Compared to single-stage detection algorithms like YOLO, Mask R-CNN demonstrates superior performance in small object detection and high-precision tasks. Previously, Li et al. employed the Mask R-CNN model to segment disease spots and insect spots on tea leaves, followed by classification using F-RNet, achieving precise segmentation and identification of the diseases and insect spots in tea leaves (45). In this study, the improved Mask R-CNN, optimized by incorporating HRNet and CBAM, can accurately locate and segment lesion regions, effectively removing background noise and preserving detailed lesion information, thus providing high-quality image data for subsequent classification.

Building on this, CNN classification models leverage their robust feature extraction and classification capabilities to perform in-depth analysis of segmented lesion regions. Different CNN models utilize their unique network architectures to extract lesion features from various perspectives, capturing rich semantic information from low to high levels and enabling fine-grained classification of complex lesion characteristics. Experimental results show that, with preprocessing by the improved Mask R-CNN, CNN models significantly improved classification accuracy for lesion characteristics, achieving efficient and accurate identification and classification of small intestine lesions in swine.

### 4.4 Shortcomings and future work

In livestock farming, early disease diagnosis faces significant challenges due to the heavy reliance on subjective and inefficient manual inspections, while laboratory methods such as PCR, though highly accurate, are time-consuming, costly, require complex procedures, and depend on specialized equipment and trained personnel. These issues are particularly pronounced in large-scale farms, often leading to the spread of epidemics and increased economic losses. Therefore, the use of AI to achieve swine disease detection can effectively break through the limitations of traditional approaches, and while ensuring accuracy, improve detection efficiency and reduce costs.

Although the multimodal diagnostic framework proposed in this study demonstrates good performance in identifying PGID, it still has some limitations. First, the current dataset is relatively small and suffers from severe class imbalance, which may limit its generalization ability in diagnosing swine diseases. To address this issue, a novel data augmentation technique was proposed, which improved model accuracy but had limited impact on class imbalance. Furthermore, due to the extremely small number of samples in certain categories (e.g., CEP), synthetic data-based augmentation and boosting algorithms also proved insufficient in resolving the class imbalance problem in this dataset. Second, the text modality enhancement relies on semantic descriptions generated by ChatGPT, which, while helpful in enhancing sample diversity, may also introduce semantic biases that could affect the stability of the text-image fusion model. These biases may manifest as inconsistencies between the generated textual features and the actual clinical presentation of the diseases, potentially leading to misalignment during multimodal feature fusion and reducing overall diagnostic accuracy.

In future work, we plan to collect a larger and more balanced dataset encompassing diverse regions and pig breeds to enhance the generalization ability of the model. To further improve diagnostic performance, more advanced deep learning architectures will be explored and compared. In particular, generative models will be considered to produce realistic synthetic samples for minority classes, thereby alleviating class imbalance and improving the robustness of the diagnostic framework. Furthermore, optimizing text enhancement is expected to improve semantic quality and credibility, with focusing on expert-annotated descriptions and domain-specific language model tuning to reduce bias and enhance multimodal robustness.

## 5 Conclusion

This study presented an intelligent diagnostic method, multimodal AI and LLMs-based classification framework, for identifying 6 types of PGID. The framework integrates a MS-TextCNN model, an improved Mask R-CNN model, CNN image classification models, and machine learning algorithms, exhibiting improved classification performance and robustness. Our results indicate that multimodal diagnostic model can significantly enhance the accuracy and efficiency in complex disease diagnosis. This study provides an efficient and accurate intelligent solution for diagnosing PGID, offering valuable reference for disease prevention and control in the livestock industry.

## Data Availability

The raw data supporting the conclusions of this article will be made available by the authors, without undue reservation.
